# Soil C and N models that integrate microbial diversity

**DOI:** 10.1007/s10311-016-0571-5

**Published:** 2016-07-21

**Authors:** Benjamin P. Louis, Pierre-Alain Maron, Valérie Viaud, Philippe Leterme, Safya Menasseri-Aubry

**Affiliations:** 1 UMR 1069 SAS, Agrocampus-Ouest, 65, Rue de Saint Brieuc, 35042 Rennes Cedex, France; 2 UMR 1069 SAS, INRA, 65, Rue de Saint Brieuc, 35042 Rennes Cedex, France; 3 Université Européenne de Bretagne, Rennes, France; 4 INRA, AgroSup Dijon, UMR 1347 Agroecology, Université Bourgogne Franche Comté, UMR Agroécologie 17, Rue Sully, BP 86510, 21065 Dijon Cedex, France

## Abstract

Industrial agriculture is yearly responsible for the loss of 55–100 Pg of historical soil carbon and 9.9 Tg of reactive nitrogen worldwide. Therefore, management practices should be adapted to preserve ecological processes and reduce inputs and environmental impacts. In particular, the management of soil organic matter (SOM) is a key factor influencing C and N cycles. Soil microorganisms play a central role in SOM dynamics. For instance, microbial diversity may explain up to 77 % of carbon mineralisation activities. However, soil microbial diversity is actually rarely taken into account in models of C and N dynamics. Here, we review the influence of microbial diversity on C and N dynamics, and the integration of microbial diversity in soil C and N models. We found that a gain of microbial richness and evenness enhances soil C and N dynamics on the average, though the improvement of C and N dynamics depends on the composition of microbial community. We reviewed 50 models integrating soil microbial diversity. More than 90 % of models integrate microbial diversity with discrete compartments representing conceptual functional groups (64 %) or identified taxonomic groups interacting in a food web (28 %). Half of the models have not been tested against an empirical dataset while the other half mainly consider fixed parameters. This is due to the difficulty to link taxonomic and functional diversity.

## Introduction

Massive use of mineral fertilisers for agricultural production has altered C, N and P cycles at the global scale. Historic global C loss due to agricultural activities is estimated at 55–100 Pg from soil C pool (Lal 1999). In Europe, the annual reactive nitrogen surplus of 9.9 Tg contributes to substantial N leaching to water bodies, volatilisation as NH_3_ and emission of N_2_O and NO (Sutton et al. 2011). Since the twentieth century, agriculture intensification has also been a major factor of land use change (Reidsma et al. 2006), and its impact is particularly noticeable for soil biodiversity (Creamer et al. 2016). A current major challenge for agriculture therefore is to adapt management practices to preserve ecological processes and reduce inputs and environmental impacts on soil, water and atmosphere.

Management of soil organic matter (SOM) is a key element of agroecological transitions because it affects both agricultural production and soil environmental functions, and conversely agricultural management affects SOM (McLauchlan 2006): SOM is highly involved in soil chemical quality by supplying nutrients for plant production, in soil physical quality by supporting structural stability and in soil biological quality by supporting biodiversity reserves (Reeves 1997). In addition to having a direct impact on the soil itself, SOM has implications in pollutant transfers to air and water. It has a strong impact on the local and global C cycles and the interest in the potential of agricultural soils to sequester C is increasing (Chambers et al. 2016). SOM dynamics and stocks partly control the emission and discharge of dissolved nutrients, such as nitrates and dissolved organic C.

Dynamics of soil C and N are greatly driven by soil microorganisms (Paul and Clark 1989; Sylvia et al. 2005). New developments in molecular biology methods provided new evidence that not only microbial abundance but also microbial diversity affects C and N cycling in the soil (e.g. Baumann et al. 2013; Juarez et al. 2013) in different experimental and agricultural contexts. Agricultural management practices interact with pedoclimatic factors and can change soil physical properties via soil tillage, soil chemical properties via organic and inorganic inputs and soil biological properties via the type of plant cultivated for instance. All three influence microbial community diversity and differences are often observed in soil microbial abundance and composition between agricultural land and less-disturbed areas such as natural areas, forest or grassland (Rampelotto et al. 2013). Other studies emphasise differences in microbial community diversity for contrasting agricultural practices: soil tillage and/or fertilisation and/or weed management (Doran et al. 1998; Feng et al. 2003; Zhong et al. 2010; Berthrong et al. 2013; Lazcano et al. 2013; Sul et al. 2013) in interaction with pedoclimatic factors (Kaisermann et al. 2013; Lienhard et al. 2013). It has been shown that microbial diversity could explain up to 77 % of carbon dynamic activities (Tardy et al. 2015). Thus, predictive tools of C and N dynamics which integrate microbial diversity should make it possible to fully evaluate the impacts of change in agricultural practises induced by agroecological transition.

Modelling of carbon (C) and nitrogen (N) dynamics is an essential tool to predict SOM dynamics in response to pedoclimatic and agricultural systems (Manzoni and Porporato 2009) to adapt relevant management practises. Today C and N dynamics models are predominantly dynamic deterministic compartment models (Manzoni and Porporato 2009), in which SOM transformation is assumed to follow first-order kinetics, and where SOM is conceptually divided into several homogeneous pools such as pool of organic matter, microbial biomass or crop residue (Halfon 1979, cited in Manzoni and Porporato 2009), each with its unique decomposition rate, and more or less resistant to decomposition. Many examples of models that integrate microbial biomass are found in the literature, which confirms the interest of including these microorganisms in soil C and N dynamics models (Schimel 2001; Neill and Gignoux 2006). However, questions about incorporating information about microbial diversity continue to enliven the debate (e.g. Nannipieri et al. 2003; McGuire and Treseder 2010; Nielsen et al. 2011; Treseder et al. 2012; Graham et al. 2014). The diversity of soil microorganisms, i.e. number of different species and relative contribution of each species to the community composition, is extensive, with a high level of functional redundancy in C and N transformations (Nannipieri et al. 2003); consequently, it is legitimate to question whether integrating diversity parameters into models could better predict soil C and N dynamics. It has long been assumed that soil microbial diversity has no functional significance because of the high functional redundancy of soil microorganisms (Nannipieri et al. 2003). This assumption was partly justified by the lack of a demonstrated diversity–function relationship, which was mainly attributed to technical limitations, which often restricted analysis of the diversity to indigenous soil microbial communities. This makes difficult to explicitly integrate the microbial community diversity in soil C and N models (Maron et al. 2011). Progress in analytical methods over the two last decades, such as in molecular biology, has generated new methods and has provided the necessary tools to reconsider this assumption (Maron et al. 2007).

In this context, the objective of this paper was to identify the various ways to further develop predictive models of C and N dynamics integrating soil microbial diversity. Focusing on the agricultural management context, we first summarise existing knowledge about the role of microbial diversity in C and N dynamics. We then analyse advantages and limits of how microorganisms have been incorporated into current models. Finally, we discuss methodological approaches towards a better integration of microbial diversity in soil C and N dynamics models.

## Role of microbial diversity in soil carbon and nitrogen dynamics

Diversity is a generic term which can be described in different ways. In this review, we refer as *taxonomic indexes*, the diversity as described by synthetic indexes taking into account a number of entities such as genotypes or species, and their relative distribution in a community: richness, evenness, Shannon index etc. To refer to the identity of the present entities as well as their abundances in the community, we used the common term *taxonomic composition*. Eventually, diversity describing functional traits of the community is referred as *functional diversity*. Considering these different ways of describing microbial community diversity, different approaches have been developed to demonstrate its role in soil carbon and nitrogen dynamics.

### Influence of reduced soil microbial diversity

Several experimental studies have enabled to assess the impact of soil microbial diversity on C and N dynamics by manipulating diversity and mainly by artificially reducing soil microbial diversity (Nielsen et al. 2011). These studies created gradients of microbial diversity in the soil while keeping a constant biomass, and measured one or several soil functions for each level of diversity. Three main methods exist, each with advantages and disadvantages (Table 1). The “construction” method is performed by creating different microbial consortia (i.e. number of species and/or taxonomic groups) by combining microbial species after isolating them in culture media (Deacon 1985). This helps understand the link between taxonomic and functional diversity by testing hypotheses of functioning in the presence or absence of specifically chosen microorganisms, such as species that use specific substrates, to study facilitation mechanisms among microorganisms (Wohl et al. 2004). The number of taxa is generally small (<100) compared to the diversity of indigenous communities, and only culturable microorganisms can be isolated, which represent less than 10 % of all existing microorganisms (Maron et al. 2011). These taxa often have particular traits such as a fast growth rates, and studies using this method mainly focus on bacterial or fungal taxa, suppressing possible interactions between them (Romaní et al. 2006). The other two methods, called “destruction” and “erosion” (Table 1), create different levels of microbial diversity by (1) eliminating different quantities of soil microorganisms through different durations of fumigation with chloroform (Jenkinson and Powlson 1976) or by (2) inoculating sterile soil with different dilutions of a solution containing microorganisms in suspension (Griffiths et al. 2000). Both approaches provide more realistic diversity levels regarding the number of taxa and coexistence of bacteria and fungi compared to the construction method. However, they also have major disadvantages or “hidden effects” (Huston 1997) represented by a possible increase in soil nutrients from dead cells in the destruction method, and the non-random selection of taxa due to the preferential elimination of rare ones in the erosion method. Reduction of diversity is assessed by measurement of taxonomic indexes, but the effect on taxonomic and functional composition is not controlled.

**Table 1:** Experimental methods used to create different microbial diversity levels

Method	References	Principles	Advantage	Disadvantage
Construction	Deacon (1985), Robinson et al. (1993), Setälä and McLean (2004), Wohl et al. (2004), Bell et al. (2005) and Tiunov and Scheu (2005)	Combinations of different microorganisms previously isolated in culture media	Taxa selection	Culture-dependency unrealistic diversity levels
Destruction	Degens (1998), Griffiths et al. (2000), and Griffiths et al. (2004)	Different duration of fumigation events	Realistic diversity levels	Hidden effect (Huston 1997): increase in soil nutrients
Erosion	Griffiths et al. (2001), Griffiths et al. (2004), Wertz et al. (2006, 2007); Baumann et al. (2013), Juarez et al. (2013) and Philippot et al. (2013)	Successive dilutions of a soil suspension	Realistic diversity levels	Hidden effect (Huston, 1997): non-random taxa selection = elimination of rare taxa

Most construction method results find a positive relationship between microbial diversity and soil processes involved in soil C and N cycles when there are fewer than 10 species (Deacon 1985; Robinson et al. 1993; Wohl et al. 2004), but they also reveal functional redundancy (Setälä and McLean 2004; Bell et al. 2005; Tiunov and Scheu 2005) in microbial communities when there are more than 10 species (except for Tiunov and Scheu 2005). The unrealistically low level of diversity obtained from the construction method can result in low niche complementarity (Loreau 2001). It is likely that certain non-cultivable microorganisms are able to perform functions that the cultivable ones cannot (Hättenschwiler et al. 2011). Genetic diversity increases functional diversity and helps microorganisms exploit a vast range of organic C compounds (Burns et al. 2013). This unattained resource partitioning could lead to an apparent redundancy in studies using complex substrates (SOM or litter) as a C resource (Setälä and McLean 2004; Bell et al. 2005), meaning that mineralisation stops because certain compounds cannot decompose or interactions (competition and facilitation) between microorganisms (e.g. bacteria vs. fungi) are not possible.

A hierarchy of functioning is often reported between different levels of diversity in more species-rich communities: the higher the level of diversity (i.e. higher richness or Shannon index), the higher the process, even if differences are not always statistically significant. We focused on nine studies based on destruction and erosion methods. Four studies (Degens 1998; Griffiths et al. 2001; Wertz et al. 2006, 2007) found no statistically significant differences in the measured processes between diversity levels and concluded that no relationship exists between microbial diversity and soil processes. The results of three studies (Griffiths et al. 2000; Baumann et al. 2013; Juarez et al. 2013) indicate a relationship between microbial diversity and soil processes such as C decomposition and denitrification, which are highly redundant functions in the microbial world (Griffiths et al. 2000), and also more specialised processes such as nitrification or methane oxidation (Griffiths et al. 2000). This suggests that decreased diversity initially might not affect soil processes; however, beyond a given threshold, diversity could become a limiting factor affecting soil processes. Another hypothesis to explain the complex relationship between diversity and soil processes is that community composition could be a determinant parameter rather than richness per se (Cox et al. 2001; Griffiths et al. 2004; Philippot et al. 2013). In this case, both diversity and community composition could have an impact on soil processes, but the specific effect of each taxa might depend on environmental conditions and biotic interactions between microorganisms (Nielsen et al. 2011) causing an increase (facilitation) or a decrease (inhibition) in functioning. Under this hypothesis, the effect of change in diversity on C and N dynamics may be more variable.

### Relationship between microbial community composition and soil processes

Recent advances in molecular biology make it possible to track and analyse specific functional genes, which provides the opportunity to compare the presence, abundance and diversity of genes in different communities (Colloff et al. 2008). This method is only possible for a few processes, most of them involved in the N cycle, such as denitrification or nitrification, because of the well-known genetic coding of the enzymes responsible for transforming N (Maron et al. 2007). For other processes, such as C decomposition, that involve highly complex and mostly unknown sets of functional genes, an approach that focuses on functional genes is highly problematic making very difficult so far to describe functional diversity. An alternative approach is to study the link between taxonomic and functional composition by determining whether taxonomic groups are related to the processes. Relationships between bacterial phyla and functional traits suggest that the phylum level has ecological coherence (Philippot et al. 2010) meaning that members of one phylum share similar life strategy or traits that make them different from members of another phylum. Certain bacterial phyla that only grow and reproduce in nutrient-rich environments (Langer et al. 2004) are classified as “copiotrophs” (Fierer et al. 2007). They also are identified as “r-strategists” or opportunists because they grow rapidly after the addition of easily available C sources in the environment and are highly competitive for this kind of substrate. Other phyla, grown in nutrient-poor environments (Langer et al. 2004), are classified as “oligotrophs” (Fierer et al. 2007). They are mainly “K-strategists” because their metabolism is slower and synthesises more complex enzymes, making possible the decomposition of recalcitrant compounds. These functional traits of phyla have been observed in different studies, such as those studying decomposition of plant residues (Pascault et al. 2010) or comparing it to that of SOM (Pascault et al. 2013) or in a N fertilisation gradient (Fierer et al. 2012). Even when these classifications are identified, the phylum remains a high taxonomic rank, meaning that members of one phylum can exhibit different physiological traits. Overall, the literature shows the importance of community composition in soil processes. However, knowledge remains limited, and further study is required to understand the influence of microbial composition on C and N dynamics and maybe identify key taxa as indicators of microbial community functional traits (Nannipieri et al. 2003).

The overall view of the cited references bring evidence that soil microbial diversity does not have a neutral effect on the soil processes involved in C and N dynamics. But we are still lacking a full understanding of the underlying changes in soil community functioning with shifts in soil diversity (Nielsen et al. 2011; Graaff et al. 2015). It seems that a change in taxonomic diversity could lead to a change in the functional traits of the whole community and thus to a change in soil functioning.

**Fig. 1:**
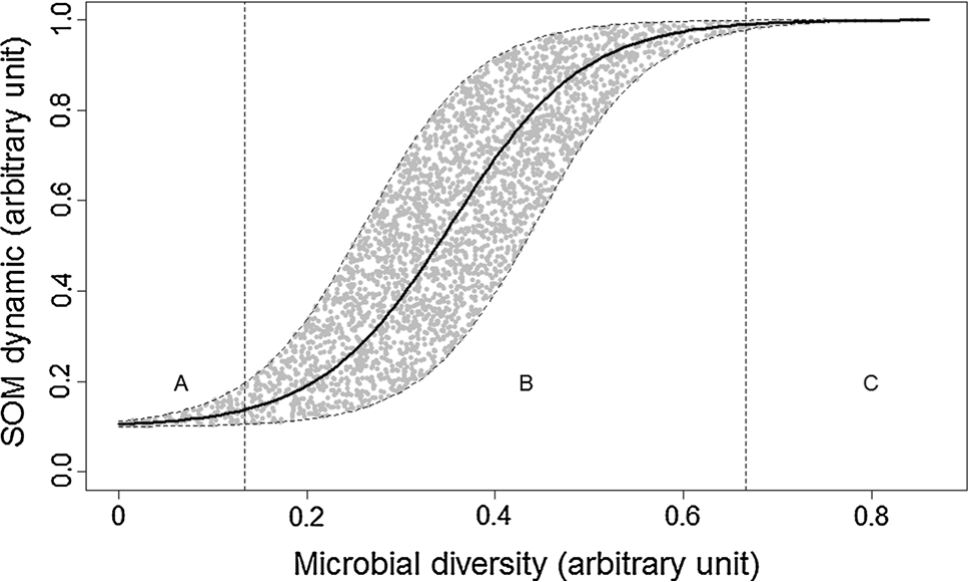
Conceptual diagram of the relationship between microbial diversity and soil organic matter (SOM) dynamics. *Solid line* general relationship; *dashed lines* area of variability in the relationship; *grey points* variability of pathways in the relationship; *A* area in which the relationship is minimum; *B* area of potential highest variability; *C* area with no relationship

To summarise, we propose a conceptual model (Fig. 1) that assumes an increase in soil functioning with increased diversity. At low levels of diversity, the redundant relationship found in construction method studies could be an artefact of the method. However, low levels of diversity have been shown to develop under real conditions when a major perturbation occurs, such as fire or severe pollution, and are often associated with a reduction in soil C and N transformations (e.g. Griffiths et al. 2000, 2001, 2004). It is likely that at low microbial diversity soil processes reach a minimum, which lies a certain distance from a potential maximum (Fig. 1, section A), but grows rapidly with an increase in diversity (Fig. 1, section B). Conversely, at high levels of diversity, we suggest that a threshold exists at which processes are not affected by an increase in diversity (Fig. 1, section C) because functional redundancy is reached or because diversity is no longer the determinant factor. This relationship is similar to the well-known mass-ratio hypotheses in plant community diversity (Grime 1998). On average (Fig. 1, solid line), an augmentation of dominant species (i.e. augmentation of diversity) controls the ecosystem functioning. However, the rate at which this plateau is reached (Fig. 1, section B) is controlled by the functional traits hypothesis (Díaz et al. 2007) which states that ecosystem functioning depends on functional diversity determined by the composition of the community. Besides, this relationship depends on the redundancy in studied processes or whether another parameter becomes more determinant. For instance, the relationship between diversity and processes seems to be expressed more significantly when substrate availability is higher (Philippot et al. 2013; Tardy et al. 2014). This suggests that the general relationship could be “situation-dependent” (e.g. substrate availability-dependent). More knowledge is needed to quantify this relationship and its potential variability in different situations.

## Soil microbial diversity in current soil carbon and nitrogen dynamics models

Models are characterised by state variables corresponding to C or N compartments and kinetics parameters. According to Manzoni and Porporato (2009) reviewing approximately 250 models of C and N dynamics developed in the last 80 years, thirty per cent of the models have no microbial compartment. Thirty per cent represent microbial biomass as an additional organic matter compartment. Among these, few models represent explicitly microbial biomass (linearly or nonlinearly) in first-order kinetics of C and N dynamics (Wutzler and Reichstein 2008). Forty per cent of the models use at least two state variables to represent microorganisms, suggesting a more explicit representation of their diversity. Although models might represent the observed reality, the initialisation of such models remains critical as each soil condition requires an optimisation analysis to initialise the OM compartments and their decomposition rates. This section focuses on the latter model group plus five other models developed since 2009.

### Representation of microbial diversity in existing C and N dynamics models

**Fig. 2:**
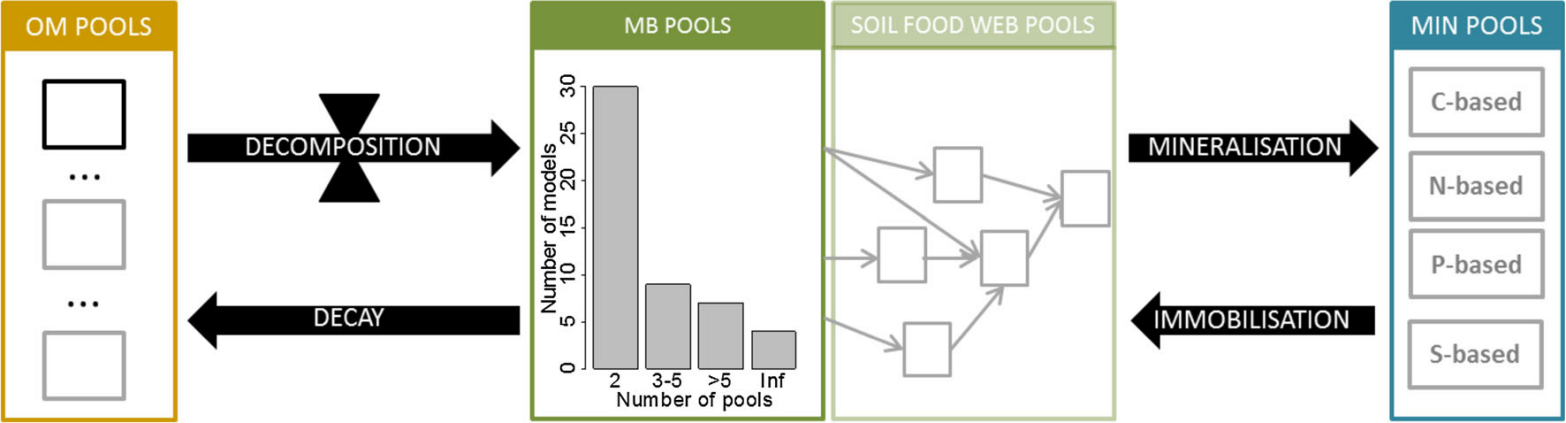
Diagram of microbial diversity in current carbon and nitrogen dynamics models. *OM* organic matter, *MB* microbial biomass, *MIN* mineral compounds. *Black* pool always encountered in models; *grey* pool specific to certain models

We distinguished two model categories (Table 2 and Fig. 2): models representing the functional diversity of soil microorganisms (FD), and soil food web models representing taxonomic diversity (TD).

**Table 2:** Classification of models representing microbial communities according to the class of microbial community (MC) representation and the number of pools associated with the class (from Manzoni and Porporato, 2009)

Model	Reference	MC	Pools
PWNEE	Patten (1972)	TD	6
PHOENIX	McGill et al. (1981)	FD	2
NCSOIL	Molina et al. (1983)	FD	2
–	Hunt et al. (1983)	TD	4
NCSOIL	Hadas et al. (1987)	FD	2
–	Hunt et al. (1987)	TD	11
–	Leffelaar (1986)	FD	2
–	Leffelaar and Wessel (1988)	FD	2
–	Robinson et al. (1989)	TD	2
DAISY	Hansen et al. (1991)	FD	2
GEM	Hunt et al. (1991)	TD	5
DNDC	Li et al. (1992a, b)	FD	4
–	Griffiths and Robinson (1992)	TD	2
–	Ruiter et al. (1993)	TD	9
Ecosys	Grant et al. (1993)	FD	4
–	Kersebaum and Richter (1994)	FD	2
Q-model	Bosatta and Agren (1994)	FD	Inf
Q-model	Bosatta and Agren (1995)	FD	Inf
Q-model	Bosatta and Agren (1996)	FD	Inf
–	Zheng et al. (1997)	TD	2
NCSOIL	Hadas et al. (1998)	FD	2
–	Zheng et al. (1999)	TD	2
–	Henriksen and Breland (1999)	FD	2
DNDC	Li et al. (2000) and Stange et al. (2000)	FD	4
SOILN-NO	Korsaeth et al. (2001)	FD	2
CANTIS	Garnier et al. (2001)	FD	2
–	Loreau (2001)	FD	m
Ecosys	Grant (2001)	FD	9
–	Kravchenko et al. (2004)	FD	2
–	Moore et al. (2004)	TD	2
–	Foereid and Yearsley (2004)	TD	2
–	Long and Or (2005)	FD	2
CN-SIM	Petersen et al. (2005a, b)	FD	2
INDISIM-S	Ginovart et al. (2005)	FD	2
–	Kuijper et al. (2005)	TD	5
–	Moore et al. (2005)	TD	10
EnzModel	Allison (2005)	FD	2
–	Fontaine and Barot (2005)	FD	2
–	Raynaud et al. (2006)	FD	2
BACWAVE-WEB	Zelenev et al. (2006)	TD	5
GDM	Moorhead and Sinsabaugh (2006)	FD	3
–	Roy et al. (2008)	FD	2
TOUGHREACT-N	Maggi et al. (2008)	FD	4
NICA	Ingwersen et al. (2008)	FD	2
CEM	d‘Annunzio et al. (2008)	FD	Inf
–	**Miki et al. **(2010)	FD	2
DEMENT	**Allison **(2012)	FD	NB
–	**Waring et al.** (2013)	FD	2
SYMPHONY	**Perveen et al.** (2014)	FD	2
	**Wieder et al.** (2014)	FD	2

References in bold were published after Manzoni and Porporato (2009). The class are FD: representation of functional diversity and TD: Soil Food Web models representing taxonomic diversity. Number of pools is equal to the infinite (Inf) for the models presenting a continuous of microbial diversity

The FD models are designed to explicitly model the diversity of substrate decomposition kinetics, by representing different affinities of microbial functional pools for substrates. Defining multiple functional pools enables multiple kinetic components in the model, such as decomposition, growth and decay rates, depending on the organic matter/microbial pool combination considered (e.g. Hansen et al. 1991; Garnier et al. 2001; Moorhead and Sinsabaugh 2006; Miki et al. 2010; Perveen et al. 2014). Functional pools are represented as discrete compartments in 89 % of the FD models. In the guild-based model developed by Moorhead and Sinsabaugh (2006), the “opportunist pool” represents microorganisms that compete highly for easily degradable material and have fast growth and decay rates when the resource is missing. The “decomposer pool” represents microorganisms that degrade cellulosic and lignocellulosic compounds. The “miner pool” represents microorganisms that degrade humified organic matter. In a more recent model, Perveen et al. (2014) distinguished two pools of microorganisms to better simulate the priming effect. The “SOM builders” pool is composed of microorganisms that degrade only fresh organic matter. The “SOM decomposers” pool represents microorganisms that degrade fresh organic matter and SOM. More detailed models with a larger number of microbial pools have been developed to represent complex interactions between functional pools or interactions between soil microorganisms and plant diversity (Loreau 2001). Grant (2001) distinguished nine microbial pools based on nine different metabolisms differing in the source of energy and nutrients mobilised; this representation enables different structural and kinetic components in substrate-microbe complexes.

TD models (28 % of those listed) distinguish taxonomic groups of microorganisms and focus on interactions along the food web. They are used to analyse contributions of these groups, specifically on N mineralisation (Hunt et al. 1987; Ruiter et al. 1993; Zheng et al. 1999) or C and N dynamics (Hunt et al. 1983; Kuijper et al. 2005). The microbial groups differ in their nutrient ratios, feeding preferences, life spans, assimilation efficiencies, production-to-assimilation ratios and decomposability.

### Advantages and limitations of existing modelling approaches

The models representing functional microbial diversity are mainly theoretical and based on conceptual microbial pools characterised by distinct functional traits.

They represent selected groups of microorganisms with distinct functional traits, mainly to test hypotheses on the processes involved in C and N dynamics. The corresponding pools might not be explicitly identified (e.g. Hansen et al. 1991) or distinguished according to known functional traits, as in the distinction between autochthonous microorganisms mainly found in environments rich in recalcitrant material and zymogenous microorganisms found in environments rich in easily decomposable material (Kersebaum and Richter 1994; Hadas et al. 1998; Garnier et al. 2001). Few models identify microbial functional pools in explicit taxonomic microbial groups. In these models, bacteria and fungi are often separate pools because their physiological differences induce contrasting C and N stoichiometries, and their relative abundance influences C and N dynamics and net primary production at the ecosystem scale (Waring et al. 2013). All food web models have explicit microbial pools because they study nutrient and energy transfer between taxonomic groups in an ecosystem. Two groups of microorganisms are found in soil food web models: decomposers, composed of bacteria and fungi, and predators of decomposers, mainly composed of protozoa and nematodes.

**Fig. 3:**
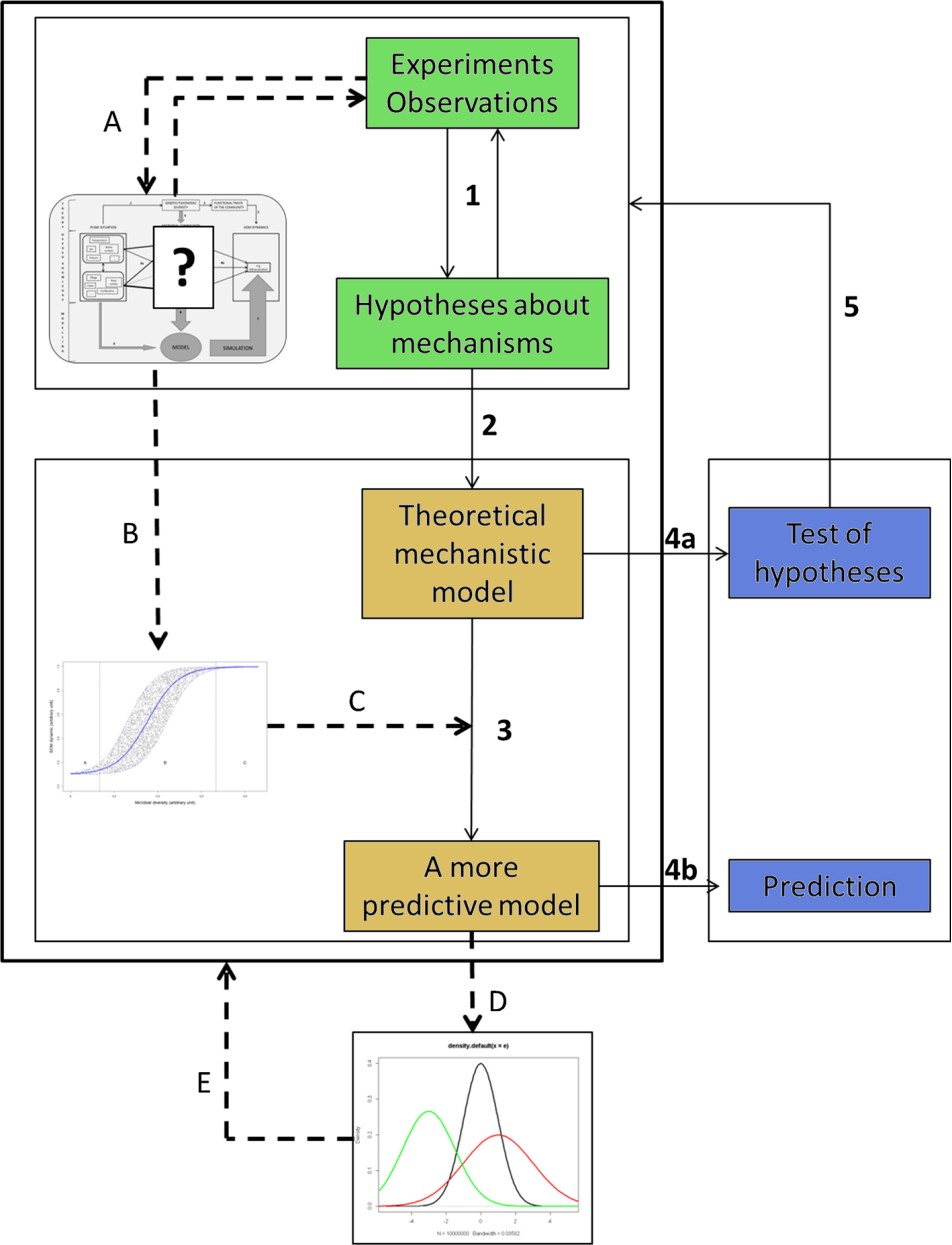
Diagram of model design (*solid arrows with numbers*) and stages for integrating microbial community descriptors (*dashed arrows with letters*). Stages correspond to (*1*) interactions between analysis of experimental/observed data that enable making hypotheses and hypotheses that influence future experiments, (*2*) translation of hypotheses into mathematical language, (*3*) model calibration and validation, (*4a*) simulations for testing hypotheses enabling (*5*) experiment/hypothesis interactions or (*4b*) for predictions, (*A*) search for best microbial community descriptors, (*B*) statistical learning, (*C*) coupling mechanistic modelling with statistical modelling, (*D*) sensitivity and uncertainty analyses, which help (*E*) in all model design

Almost half of the models that consider microbial diversity (FD and TD) have not been assessed with empirical datasets, i.e. data from an experiment or from field observations, at least in the reviewed articles. These heuristic models are essentially used to test hypotheses (Fig. 3, arrows 4a and 5) about the coexistence of species in heterogeneous soil (Long and Or 2005) or effects on C and N dynamics of species and enzyme diversities (Allison 2012), rhizodeposition and microbial interactions (Robinson et al. 1989; Griffiths and Robinson 1992; Foereid and Yearsley 2004), global change (Kuijper et al. 2005) or anthropogenic and natural disturbances (Moore et al. 2005). Some were only analysed at equilibrium to assess the model’s capacity to simulate processes such as the priming effect (Perveen et al. 2014), plant-soil feedback in plant communities (Miki et al. 2010), plant persistence and C storage (Fontaine and Barot 2005), or the effect of microbial diversity on ecosystems (Loreau 2001). The main reason not to evaluate such models by comparing predicted and observed data is that they are in essence theoretical; therefore, data are not available to calibrate the parameters (e.g. Moorhead and Sinsabaugh 2006). At most, the realism of model behaviour is assessed by comparing model outputs to general patterns from the literature or to those observed in real situations. These models are appropriate tools to assess ways to translate a hypotheses or a process into mathematical language, but are less suitable for prediction in the current state of knowledge (Fig. 3, arrow 4b). The main exceptions are models focusing on N dynamic to whom genes implicated in the different processes are well known and where microbial biomass can be subdivided in measurable functional groups involved in these processes such as nitrifiers and denitrifiers (Leffelaar 1986; Leffelaar and Wessel 1988; Li et al. 1992a, b, 2000; Ginovart et al. 2005; Raynaud et al. 2006; Maggi et al. 2008).

Other models were evaluated by comparing their predictions to experimental data (Fig. 3, arrow 3). However, a limit to these models is that initial values and parameters related to microbial communities are often assumed fixed or calibrated values. For instance, the initial biomass of each model compartment is often non-measurable. When compartments represent known taxonomic groups (e.g. bacteria vs. fungi; most compartments in TD), the initial biomass of each group is measurable, but when compartments represent conceptual functional groups, theoretical values are assumed. In the first case, compartments represent a high level of taxonomic classification, and we question whether this subdivision is always sufficient. This representation is often used at an ecosystem scale, but at smaller scales it may be relevant for better explaining bacterial diversity. In the second case, initial values are most often defined from the literature (Leffelaar and Wessel 1988; Li et al. 1992b; Henriksen and Breland 1999; Korsaeth et al. 2001; Foereid and Yearsley 2004; Ginovart et al. 2005), but they can also be fitted from a control incubation (Hadas et al. 1998) or estimated during model calibration (Maggi et al. 2008). The main issue of using assumed values compared to real measures is that making correct assumptions is not guaranteed, and the values may not be accurate. Yet, this information can be of great value. For instance, sensitivity analysis showed that the fraction of the late-stage decomposer pool in initial microbial biomass at time *t* = 0 in the model of Ingwersen et al. (2008) has the highest sensitivity among all fitted parameters. However, authors rarely discussed this aspect, and the way initial values are chosen is not always documented.

Efforts have been made to more explicitly represent microbial communities in C and N dynamics models, but the overview of the existing models shows that they multiply theoretical or conceptual functional compartments of microorganisms. This approach comes with difficulties in getting data to evaluate the models, creating a lack of predictive models.

## What way towards predictive modelling approaches including soil microbial diversity?

### Getting additional datasets to inform and improve explicit models

As mentioned above, a lot of data are available on C and N dynamics and microbial diversity in a lot of context (different soil types, studied functions, used substrates etc.). Metaanalyses could enable to summarise these results and find general relationships between microbial diversity and C and N dynamics. However, considering the differences in the protocols measuring soil microbial diversity, deriving generic quantitative relationships between soil microbial diversity and C and N dynamics suitable to calibrate models remain challenging (Graaff et al. 2015). Consequently, additional datasets are still necessary (1) to further deepen our knowledge of soil microbial diversity impact on C and N dynamics, (2) to improve and evaluate the current models by testing with numerous quantitative diversity—C,N dynamics relationship, (3) and eventually provide more predictive modelling approaches.

To develop models with a relatively wide validity domain, these datasets must include data on soil microbial communities covering a large range of diversity and presenting enough intermediate levels to capture the likely nonlinearity of the relationship (Fig. 1). The data must be representative of a range of pedoclimatic conditions and agricultural systems. As reviewed in “Role of microbial diversity in soil carbon and nitrogen dynamics” section, the aim of experimental studies creating a gradient of diversity is to detect potential difference of functioning when the diversity decreases. Consequently, they work with few levels of diversity (around 5) and seldom if ever build a relationship between diversity descriptors and soil functions. We suggest that the number of levels should be doubled (around 10) and range between a high rate of decrease and a non-decrease in the diversity. In addition, in these incubation experiments determination of microbial diversity should not be restricted to a unique initial point in time (as often done), but should be assessed parallelly to functions measurements since both are known to be strongly dynamic variables.

As microbial diversity is highly impacted by soil physical and chemical properties, climate and land use (Maron et al. 2011), the natural gradients of diversity found in soils could be used to study the relationship between diversity and C and N dynamics (Tardy et al. 2015). The major limit is the unavoidable presence of confusion between factors influencing C and N dynamics. Being capable of determine if different dynamics is a direct consequence of the gradient or an indirect consequence of the reason of the gradient is not that obvious. For instance, Rousk et al. (2010) found a strong link between a pH gradient and microbial diversity. In this case, it would be difficult to separate the biological effect (microbial diversity) from the chemical effect (pH) on C and N dynamics. However, using natural gradient, with uncontrolled factors, remains complementary to created gradient where others factors can be controlled.

In most of the available datasets based on incubation under controlled conditions to link soil microbial diversity to C and N dynamics, only the total mineralisation and decomposition fluxes (e.g. total CO_2_ emissions) are measured. We argue that the use of isotopic labelling technics and the implementation of experiments, in which soil samples will be incubated with or without added labelled plant residues, are of high interest to better understand the processes driven by microbial diversity and quantify the relationship between microbial diversity and each C and N flux (decomposition, basal respiration, priming effect). These technics combined with microbial diversity characterisation are still used in ongoing research projects (ANR DIMIMOS ANR-08-STRA-06, and ANR MOSAIC ANR-12-AGRO-0005) and should provide key datasets to further develop and parameterise mechanistic models.

### A complementary modelling approach based on taxonomic indexes

Although studies about the relationship between microbial diversity and SOM dynamics compute synthetic taxonomic diversity indexes (e.g. richness, Shannon index), none of the reviewed models use those available indexes. This can be explained by the lack of understanding of the relationship between taxonomic diversity and functional traits of a community (Heijden and Wagg 2013). Given the potential and availability of taxonomic indexes, additional effort is needed to better understand this relationship and assess the relevance of using them in predictive models.

As shown above, considering microbial diversity in C and N dynamics models significantly increases their complexity and consequently almost restricts their applicability to theoretical issues. While adding compartments helps represent functional diversity, it is limited by the exponential growth in the number of parameters introduced and the quantity of data needed to calibrate them. The resulting models with many compartments would be conceptually interesting but not highly applicable because it would be challenging to calibrate them. For prediction purposes, alternative modelling approaches based on empirical functions could be tested to account for impacts of microbial diversity in C and N dynamics models, even non-explicitly.

**Fig. 4:**
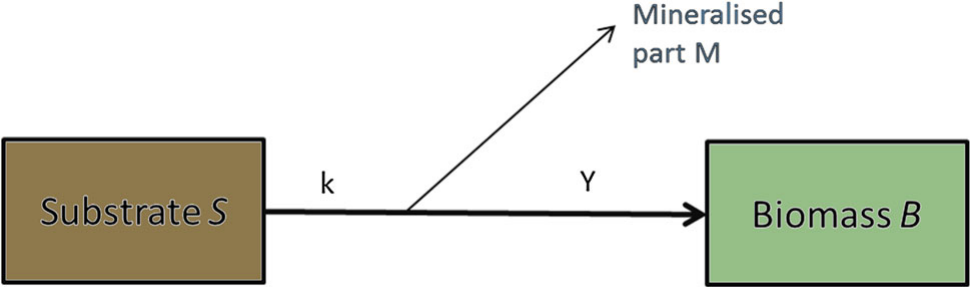
Diagram of a simple model of decomposition of a substrate

One possible alternative approach would consider soil microbial diversity descriptors in a function that modifies parameters in the first-order kinetics equation describing SOM decomposition/mineralisation: for example, simple representation of decomposition of a substrate by microbial biomass (Fig. 4). Microbial biomass would be represented by a state variable and its activity by two parameters: decomposition rate (*k*) and efficiency yield (*Y*) (Fig. 4).

The decomposition rate and efficiency yield can be corrected by functions* f* and* g*, respectively, depending on groups* θ* and* θ*′ of key taxonomic indexes, respectively:

$$ \begin{array}{*{20}l} k \hfill & {\mathop \to \limits^{         f\left( \theta \right)            } } \hfill & {k_{\text{corr}} } \hfill \\ Y \hfill & {\mathop \to \limits^{{         g\left( {\theta^{\prime}} \right)            }} } \hfill & {Y_{\text{corr}} } \hfill \\ \end{array} $$

This approach is similar to the traditional method of including the effect of pedoclimatic factors such as temperature, soil moisture or soil texture. Parameters are a function of pedoclimatic factors mainly in a multiplicative form (e.g. Hansen et al. 1991; Garnier et al. 2001). This illustrates why soil microbial communities, represented by the groups of taxonomic indexes, should be quantitatively linked to SOM dynamics, represented by decomposition rate and efficiency yield (Fig. 3, arrow B).

Since many methods measure descriptors of microbial communities and knowledge about how the latter might influence these parameters is lacking, such empirical functions are difficult to define. The need exists for better integration of empirical knowledge through statistical learning in models (Fig. 4, arrow C). In particular, statistical tools have three advantages:

1. Many exploratory tools exist (e.g. clustering, tree-based method, factorial analyses) to help identify descriptors of microbial communities that control processes of soil C and N dynamics and that are faster than mechanistic modelling and hypothesis testing.
2. Statistical modelling can be useful to link model parameters such as the decomposition rate to microbial community descriptors. Considering current limited knowledge, tools with little a priori (e.g. generalised additive models) are useful to understand complex systems in ecology and help in exploring underlying mechanisms (Guisan et al. 2002).
3. Statistical modelling comes with estimated uncertainties, which could be incorporated into traditional models by developing stochastic models, which have demonstrated their relevance for ecological modelling (Clark 2007; Katul et al. 2007).

### Uncertainties and sensitivity: assessing for progressing

Assessing the accuracy of predictions should include comparing model predictions to independent observed data. The relevance of methods used to represent microbial communities more explicitly should be assessed with sensitivity and uncertainty analysis tools (Fig. 3, arrow D), which are not used sufficiently in models of C and N dynamics. They have demonstrated their ability to assess the behaviour of complex dynamic system models, such as those simulating ground temperature (Gubler et al. 2013) and soil moisture (Wolf et al. 2008) or in some C and N dynamics models (Hunt et al. 1987; Raynaud et al. 2006; Roy et al. 2008). Sensitivity analysis can improve models and adapt microbial descriptors to include different scales of study (from micro- to global scales), mechanisms or systems (Fig. 3, arrow E). These tools could be a way to improve the generality of a model (sensu Levins 1966) by adapting or modifying it according to the situation rather than developing a model applicable to all situations. By combining these tools, experiments would help develop models, and sensitivity analysis would help design experiments.

## Conclusion

From our review, the increasing use of advanced tools from microbiology and molecular biology appears to provide evidence of the influence of microbial diversity in SOM dynamics. This diversity is driven by the combination of edaphic factors and agricultural management practices, suggesting that better understanding of its effects on SOM dynamics and its integration into models could increase the accuracy of the latter. Our review shows that a body of modelling studies already confirms that considering microbial diversity in C and N dynamics models should improve the ability to accurately predict SOM dynamics. Microbial diversity is mainly incorporated by adding new biomass compartments to represent groups of microorganisms. These studies allowed the development of mechanistic models that explicitly represent soil processes. They are needed to test new scientific hypothesis and help understand underlying mechanisms and the impact of the environment and human activity. With certain exceptions, the mechanistic approach seems to be the only one used to model soil C and N dynamics in recent decades. However, they are essentially theoretical and are not operational for prospective simulation. Combining the mechanistic approach with statistical tools could facilitate incorporation of explicit taxonomic diversity indexes and improve models that simulate real situations, which should follow a realistic quantitative approach to provide predictions conditioned by real data.
